# Safeguarding Anesthesia

**Published:** 1913-07-15

**Authors:** C. S. Tuttle

**Affiliations:** Philadelphia, Pa.


### SAFEGUARDING ANESTHESIA.

BY C. S. TUTTLE, D.D.S., PHILADELPHIA, PA.

The principal points which I shall endeavor to bring before you this evening are:

To *whom* is it safe to administer an anesthetic?

What anesthetic shall be selected to produce the *best* results with the *smallest* risk?

Frequently I have asked members of our profession who have been administering nitrous oxid for a number of years, "How do you determine an unfit subject for anesthesia?" Their replies have been many and varied. A fair example is, "If a patient is able to walk up the steps to my office without prostration I consider him fit." But in case of accident I fear this would not justify the error of judgment. Again, many men are timid and hesitate to administer a general anesthetic, and yet I have often seen these same men produce their hypodermic needle and inject cocaine into the gingival tissue, a procedure which I consider far more dangerous than the administration of nitrous oxid, somnoform or ether.

The safety in administering an anesthetic, either by a physician or a dentist, depends upon his possession of the requisite knowledge, his skill and his experience. Without these factors we forfeit our opportunity not only of relieving our patients from a maximum amount of pain and subsequent shock, but we waste a vast amount of time, for the value of general anesthetics to the dental surgeon as a time-saver has been fully demonstrated, but seems not to be fully appreciated.

What type of patient responds best to an anesthetic?

It has been my experience (and I keep an accurate record of all administrations) that the bilious and sanguine types respond best to nitrous oxid and oxygen. Analgesia in these two types is readily reached and may be maintained free from hysterical manifestations. Patients of the nervous type are prone to hysteria, and in many instances it becomes necessary to discontinue the anesthetic. Patients of the lymphatic type are very apt to have laryngeal spasm, are poor breathers, and this is one type in which I strictly avoid anesthesia.

It is remarkable to all who use anesthetics in their daily practice how easily and quietly a thin, puny patient, often of impaired health, takes an anesthetic, especially when carrying him only to the stage of analgesia. He seems to derive all the benefits, and returns to complete consciousness well and tranquil, while the more robust patient is very apt to cause us untold trouble.

In deciding upon your patient it is well to keep these points in mind: A good steady pulse, from 70 to 75, and a normal blood pressure, from 110 to 130, varying according to age of patient, when the anesthetic is started, is requisite. The first effect of an anesthetic is to send both the pulse and pressure sailing upward; but the healthy patient taking nitrous oxid and oxygen rapidly recovers normal pulsation, especially as the percentage of oxygen is increased.

What constitutes danger in the administration of an anesthetic?

It is a grave mistake to think that because the heart is strong there can be no other danger present. Luke says:

"In 90 per cent. of fatalities from chloroform the heart was found at the post-mortem to be normal." Heart disease is uppermost in many minds and is a factor to be well looked into; but it often does not prove as dangerous as the different forms of lung trouble. The inability to breathe correctly is bad, and failure to breathe demands immediate attention. If this occur the anesthetic must be removed at once and the lips rubbed from side to side briskly with a towel, which usually has the desired effect. If further trouble is manifested, artificial respiration must be resorted to quickly. Accidents from the hypodermic use of cocaine are much more perilous than those from the administration of nitrous oxid or ether. In the former the difficulty is due to the intrinsic danger of the drug; but in the latter from a disregard of danger signals. When you find the pulse rate crossing the pressure rate it is time to be on the alert. If the anesthetist does not exercise proper care of his patient at this point he is disregarding a danger signal. It seems unnecessary to submit an apparently healthy patient to any lengthy examination of the heart, which would, in many instances, have a tendency to alarm him, when about to administer nitrous oxid to bring the patient to the analgesic stage provided it is safeguarded with a combination of oxygen. But it is necessary that we obtain the blood pressure. There is nothing which gives the anesthetist more comfort than to be fully assured that the circulation is correct and that respiration is free and unobstructed.

The general anesthetics of interest to the dental profession in the order of their relative safety, are nitrous oxid and oxygen in combination, nitrous oxid, ether and somnoform. It is unnecessary to go into lengthy detail, suffice to say that I have had experience with all of them. Before the advent of our gas-mixing apparatus I frequently administered ether in cases where caries was to be removed from sensitive teeth. Giving preference to nitrous oxid and oxygen when I am the operator, I like somnoform when I am the patient. It may be of interest to relate my last experience with the latter (I have only taken it twice). I was the subject of a clinic on November 10 last, held at my father's office, he being the operator. Dr. C. H. McCowan, of West Chester, administered the somnoform. Dr. Francis A. Faught took the blood pressure. The operation consisted of the removal of caries from sensitive cavities at gingival margins. The DeFord inhaler was used. After it had been adjusted and a capsule broken the indicator was opened sufficiently to allow only a trace of somnoform to escape. With the first two or three inhalations sensation in the finger tips was lost. At this stage a tingling sensation was present over the entire body. At my suggestion tests on the cavities were made and found to be still quite sensitive. More somnoform was allowed to flow and drilling commenced. The anesthetic was then turned off, but it was soon found that more was necessary. It was also noted that a continual flow of somnoform, mixed with air, was better, in my case at least, than the start-and-stop method. The most pleasing feature of the procedure was a lack of sensation in the dentine, which lasted for some time after the anesthetic was withdrawn. This was demonstrated by repeated tests with sharp excavators and some final touches with a bur. During the early stage I could distinctly feel the pressure of the sphygmomanometer on my arm; but this soon disappeared, although I could see Dr. Faught at work. A partial obtundant effect lasted a full half hour. Aside from this not unpleasant sensation I was normal and should never have known an anesthetic had been taken. There was no nausea present, neither was there the first time I took somnoform, although it was then taken immediately after a hearty meal.

There is one minor feature, perhaps worthy of consideration, which may confront the anesthetist of shorter experience and that is timidity. Should he show signs of this, or of nervousness, he will find it quickly transmitted to his patient, with dire results. Patients in the stage of analgesia are bordering on the second stage of anesthesia, and it requires splendid technic to prevent them from passing over the border line. Close attention, a continual line of small talk, assuring the patient of safety, and, occasionally, a sharp command to prevent his giving trouble, are absolutely necessary. In other words, mental suggestion is a valuable adjunct and is acquired only by experience.

Care and judgment on the part of the anesthetist are most essential, particularly in his selection of the anesthetic to be administered. For safety, as I have said, nitrous oxid and oxygen heads the list. In American statistics recently given in the *American Medical Journal* I find the following:

"There have been no deaths from nitrous oxid and oxygen between the years 1905 and 1911, although 8,585 administrations were recorded; ethyl chlorid has 905 administrations, with no deaths reported; ethyl chlorid, ether sequence, 4,331 administrations, with one death; ether, 157,453 administrations, with 28 deaths to report." Somnoform was conspicuous by its absence of report.

In conclusion allow me to repeat that if you have a normal blood pressure and pulse rate before the anesthetic is begun, and carefully attend to the respiration, during the administration, there is small chance of trouble.—*Dental Brief.*

